# Exploring the emergence and evolution of population patterns of leisure-time physical activity through agent-based modelling

**DOI:** 10.1186/s12966-018-0750-9

**Published:** 2018-11-19

**Authors:** Leandro M. T. Garcia, Ana V. Diez Roux, André C. R. Martins, Yong Yang, Alex A. Florindo

**Affiliations:** 1 UKCRC Centre for Diet and Activity Research (CEDAR), MRC Epidemiology Unit, University of Cambridge School of Clinical Medicine, Cambridge, UK; 2 University of Sao Paulo School of Public Health, Sao Paulo, Brazil; 3 Urban Health Collaborative, Drexel University Dornsife School of Public Health, Philadelphia, USA; 4 University of Sao Paulo School of Arts, Sciences and Humanities, Sao Paulo, Brazil; 5 University of Memphis School of Public Health, Memphis, USA

**Keywords:** Physical activity, Theoretical models, Computer simulation, Complex systems, Agent-based modeling

## Abstract

**Introduction:**

Most interventions aiming to promote leisure-time physical activity (LTPA) at population level showed small or null effects. Approaching the problem from a systems science perspective may shed light on the reasons for these results. We developed an agent-based model to explore how the interactions between psychological attributes and built and social environments may lead to the emergence and evolution of LTPA patterns among adults.

**Methods:**

The modeling process consisted of four stages: (1) conceptual model development, (2) formulation of the agent-based model, (3) parametrization and calibration, and (4) consistency and sensitivity analyses. The model represents a stylized community containing two types of agents: persons and LTPA sites. Persons interact with each other (proximal network and perceived community) and with the built environment (LTPA sites) over time. Decision-making is based on the person’s intention to practice LTPA, conditioned to the perceived environment. Each iteration is equivalent to one week and we assessed a period of 10 years.

**Results:**

The model was able to reproduce population temporal trends of intention and LTPA reported in the literature. Sensitivity analyses indicated that population patterns and trends of intention and LTPA were highly influenced by the relationship between a person’s behavior in the preceding week and his current intention, the person’s access to built and social environment, and the density of LTPA sites.

**Conclusions:**

The proposed agent-based model is suitable to explore the emergence and evolution of LTPA patterns among adults, considering the dynamic interaction between individuals’ psychological attributes and the built and social environments in which they live. The model is available at 10.17605/OSF.IO/J2KAS.

**Electronic supplementary material:**

The online version of this article (10.1186/s12966-018-0750-9) contains supplementary material, which is available to authorized users.

## Introduction

There have been significant efforts to promote physical activity in both the leisure and transportation domains [1–4]. However, systematic reviews and meta-analyses [5–11] have revealed that population-based initiatives have produced small, null, or even inconsistent effects. Part of the problem is that most of these initiatives focused on individual behavior solely and did not consider the dynamic relations among individuals and between individuals and their environments [12].

Despite the accumulated knowledge on what encourages or prevents people from being physically active [12], little progress has been observed on increasing population levels of physical activity. One of the reasons is that knowledge and action on the determinants of individual behavior do not always lead to population behavior changes [13]. Leisure-time physical activity (LTPA) exemplifies this situation. Evidence about plausible determinants and interventions to change LTPA levels has grown exponentially [14]. However, and despite the growing investment in population-based initiatives to promote LTPA in the last decade, temporal trends of LTPA largely remained stable or increased marginally [15–22].

Kohl and collaborators [12] advocate the use of systems science methods to address this mismatch and widen our understanding on population-level physical activity. Under the systems science perspective, population patterns and changes of physical activity cannot be inferred only by aggregating individual behaviors. These patterns and changes derive from a complex adaptive system, composed of dynamic interactions among heterogeneous elements, which are, at the same time, autonomous and interdependent. Incorporating the systems science framework would help researchers and practitioners to understand how population patterns and trends of physical activity emerge and design effective interventions.

One method for modeling complex adaptive systems is agent-based modeling. In this modeling approach, a system is represented as a composition of autonomous entities, called agents, and the environment in which they live. Each agent possesses decision-making capabilities, determined by a set of rules on how to act in face of the agents’ interaction with the surrounding environment and other agents [23, 24]. Agent-based modeling has been increasingly utilized to investigate how dynamic processes involving built and social environments affect population health [25], including health behaviors and chronic diseases [26, 27].

Reasons to develop an agent-based model of population patterns of LTPA are fourfold. First, LTPA is consistently associated with longevity and better health [28] and is one of the pillars for most population-based physical activity promotion initiatives [29]. Second, agent-based models can help researchers understand the interrelations and impacts of factors at different levels — from individual-level factors to environments and policies — thus facilitating the identification of innovative ways to intervene. Third, all agent-based models related to population levels of physical activity published so far focused on transport-related physical activity [27]. Finally, literature and data on LTPA are vast and rich, in quantity and range, providing a solid modelling foundation.

Our goal was to develop an agent-based model that can allow researchers to explore the emergence and evolution of population patterns of LTPA among adults, taking into consideration the interaction between individuals’ psychological attributes and the built and social environments in which they live.

## Methods

The modeling process was composed of four stages: (1) conceptual model development, (2) formulation of the agent-based model, (3) parametrization and calibration, and (4) consistency and sensitivity analyses.

### Conceptual model development

The conceptual model depicts the main psychological and environmental (built and social) elements and processes that may play a role in the emergence and evolution of population patterns of LTPA in adult populations.

A detailed account of the conceptual model development is available elsewhere [30]. First, we drafted a version based on the expertise of all authors. Second, we iteratively updated the conceptual model using information obtained from a literature review on psychological attributes, and built and social environments related to LTPA. Third, an intermediate version of the model was assessed by 18 experts in at least one of the topics approached by the conceptual model. The final version of the model integrated expert assessments and additional information found in the literature.

**Fig. 1:**
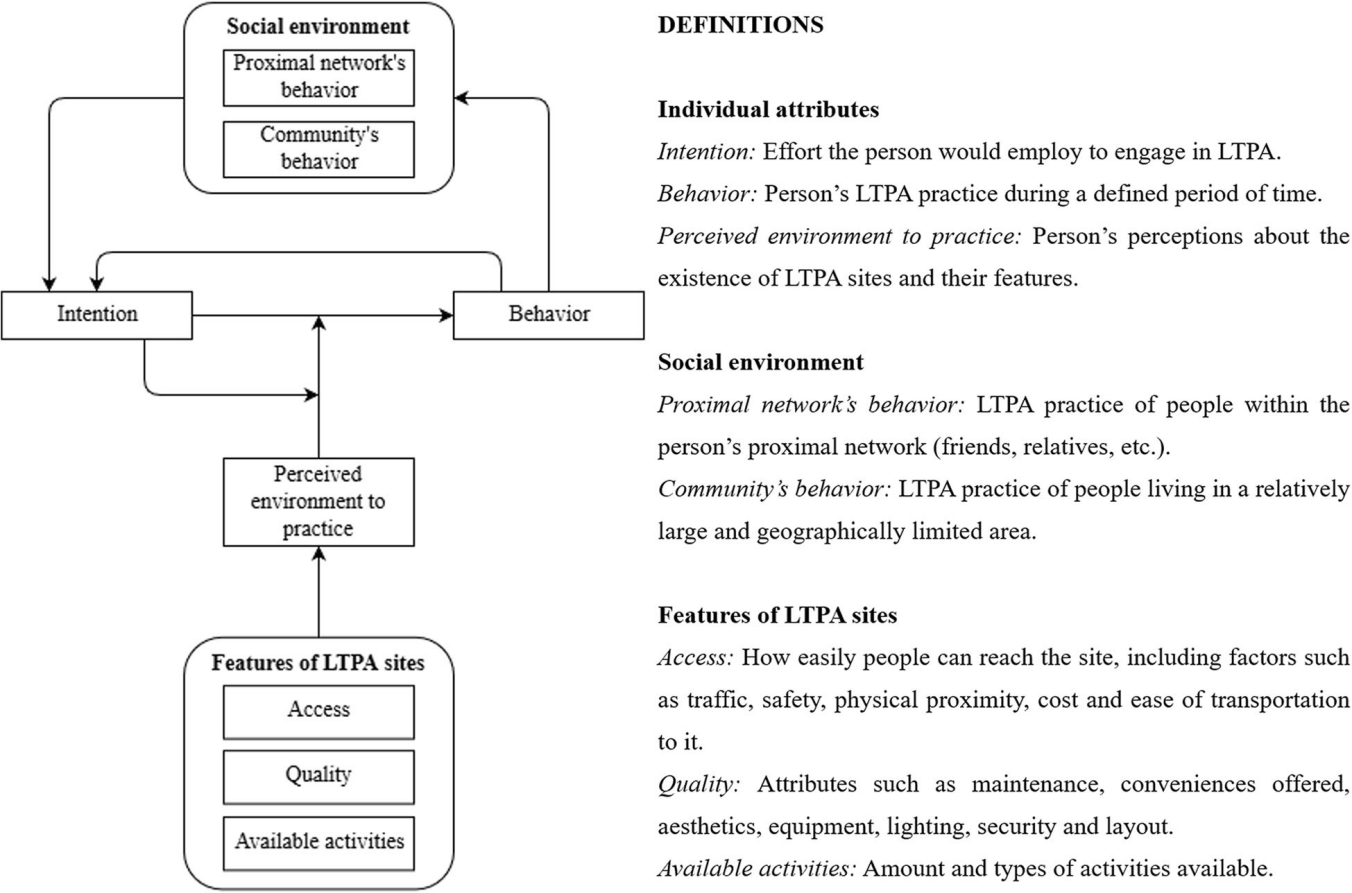
Conceptual model. Complete description published elsewhere by Garcia et al. [30]. LTPA: leisure-time physical activity. Arrows indicate the direction of influence

Figure 1 summarizes the conceptual model. We did not intend to produce a comprehensive framework of factors influencing physical activity behavior but a rather focused one to inform and support the development of the agent-based model, which was delimited a priori to encompass the main psychological and environmental elements and processes shaping population patterns of LTPA. Therefore, some aspects, such as demographic attributes, were not included as we felt they were not highly relevant to our current research questions.

### Agent-based model

The agent-based model was developed based on the conceptual model. We used the Overview, Design Concepts, and Details + Decision (ODD+D) protocol [31] to formulate the structure and content of the agent-based model. Below we present a brief description of the model main aspects. For the full description, see Additional file 1.

#### Overview

The model has two types of agents: persons and LTPA sites. A person has four attributes: location, intention (ranging from 0.03 to 0.97), one favorite type of LTPA (among 10 available in the model), and behavior (did or did not practice LTPA in the previous week). An LTPA site also has four attributes: location, quality (ranging from 0 to 1), and number and types of activities available (among 10 available in the model).

A square grid (50 × 50 patches as default) with non-contiguous sides represents the physical space, not emulating any particular location or community. Patches are characterized as being or not a LTPA site. By default, the model contains two thousand people. Persons are placed in the grid in patches that are not LTPA sites. Time is discrete, and each iteration is equivalent to one week, a timeframe frequently used to investigate physical activity behavior and that, therefore, makes easier to calibrate and compare our model against empirical data. We assessed a period of 10 years (520 iterations).

Every week, each person’s intention is updated based on his behavior and the behavior of his proximal network and perceived community in the previous week. Then, each person decides whether he will practice LTPA during the current week, based on the new level of intention and conditional to the perceived built environment. The entire process is synchronic (i.e., every person updates the same attributes at the same time) and happens in a weekly basis.

#### Initialization

At the initialization, a proportion of patches is defined as LTPA sites and randomly positioned in the grid. Each site gets a quality score drawn from a normal distribution. Next, the number of LTPAs available in each site is drawn from a uniform distribution. Then, the types of LTPAs available in each site are randomly drawn from a list of 10 possible activities using a uniform distribution.

Persons are placed randomly over patches that do not represent LTPA sites. Every person sets, randomly, none or one of the 10 available LTPA types as his favorite, drawing from a uniform distribution. Next, each person incorporates in his memory a list of LTPA sites within his perception radius — a delimited area within the grid that the person can observe — and their attributes:

- Site’s quality;
- Access (comprising factors such as traffic, safety, physical proximity, cost and ease of transportation to it), which is represented in the model as the Euclidian distance between the person and the site (the longer the distance, the worse the access);
- Whether his favorite LTPA is available at the site.

Each person then calculates and stores in his memory the sites’ perceived utility, obtained by Eq. 1:

1 $$ {u}_{s,i}={v}_{i,s}\ \left(\frac{q_s}{3}+\frac{1}{3{z}_{i,s}}+\frac{m_{i,s}}{3}\right) $$

In which *u*_*s,i*_ is the utility of site *s* as perceived by person *i*, *v*_*i,s*_ is the subjective assessment (scaling factor) given by person *i* to site *s*, *q*_*s*_ is the quality of site *s*, *z*_*i,s*_ is the access of person *i* to site *s*, and *m*_*i,s*_ shows whether the site *s* offers the favorite LTPA of person *i*. A subjective assessment scaling factor is assigned to each person–LTPA-site dyad by drawing from a normal distribution.

Next, the level of intention is set for every person by drawing from two uniforms distributions, each one applied to one of two subsets of the population, split randomly. This procedure allows more flexibility for initializing the population distribution of intention. For this work, the first subset encompasses 25% of the population and has level of intention ranging from 0.03 to 0.30, whereas the level of intention of the other 75% ranges from 0.31 to 0.97.

Each person is also assigned a behavior (i.e., did or did not practice LTPA in the past week), with probability equal to his intention. Persons who do not have any LTPA site within their perception radius are assigned to not having practiced the behavior in the past week.

Finally, a proximal network and a perceived community are defined for each person, as follows:

- Proximal network: represents those with whom the person has close relationships (such as friends and relatives). The network is formed in a two-stage process. First, the *k* closest persons in the grid are selected. Then, each link has a probability *p*, set by the modeler (0.15 for this work), to be exchanged for a link with any other person outside the initial proximal network. This procedure aims to ensure that the proximal network is mostly formed by people with similar social and environmental influences, but with some degree of variability.
- Perceived community: formed by those people within the person’s perception radius. Each person infers the social norm of the entire community looking at the behavior of the perceived community.

**Fig. 2:**
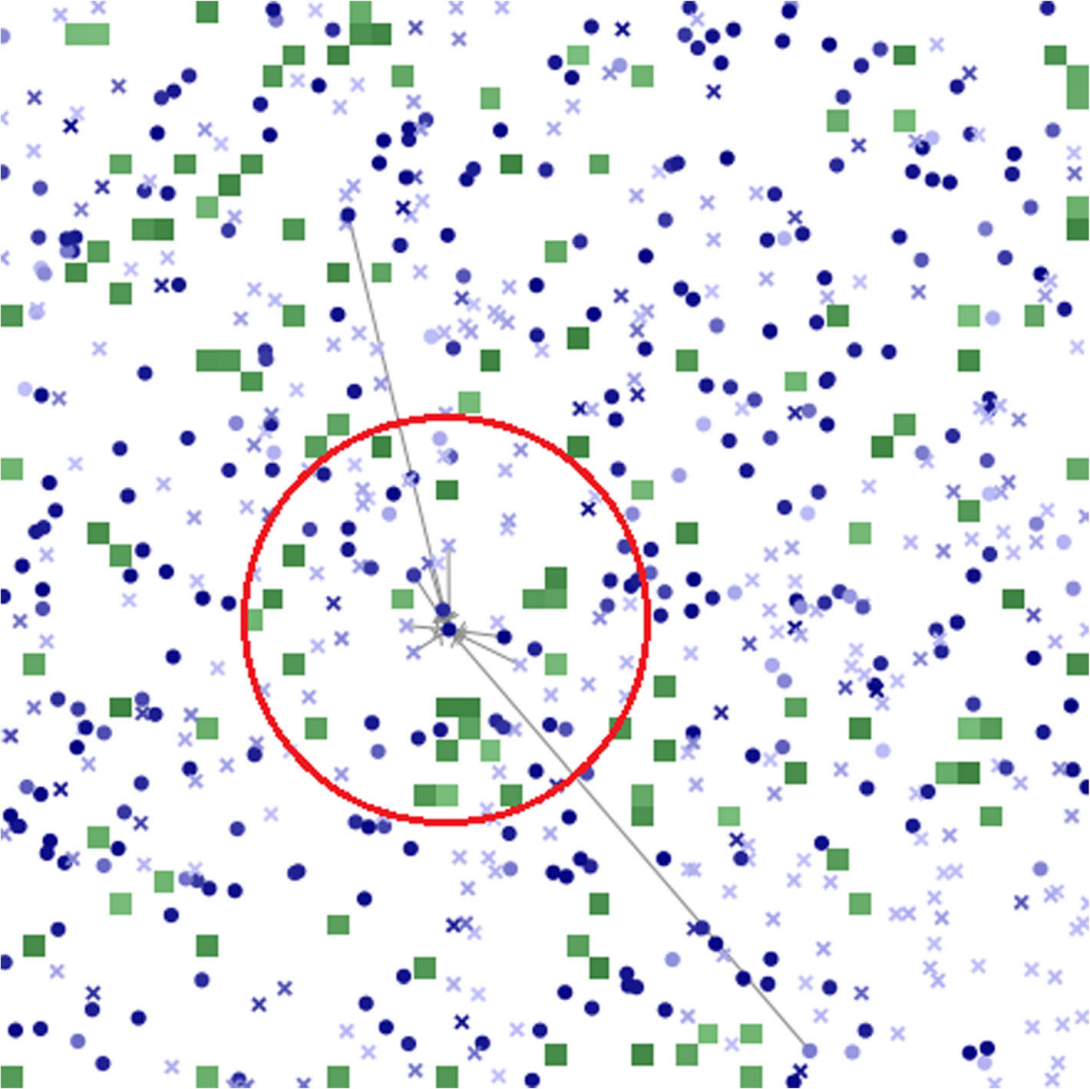
Example of a grid after initialization. Green patches are leisure-time physical activity (LTPA) sites. The darker the green, the higher the sites’ quality. Persons are indicated by crosses (did not practice LTPA in the previous week) and circles (practiced LTPA in the previous week). The darker the blue, the higher the person’s intention. Gray lines represent the person’s proximal network and the red circle his perception radius

Initial configurations of the same scenario may differ between replications as they mostly rely on stochastic processes. Figure 2 exemplifies a grid after the initialization.

#### Changes over time

In our model, persons tend to maintain their habitual intention while seeking to adapt it according to the behavior observed from their proximal network and community. Moreover, the translation of intention into behavior is conditional to the perceived built environment.

The person’s decision-making process is grounded in an extension of the Continuous Opinions and Discrete Actions model [32], which posits that a discrete action (in this case, practicing LTPA or not) is a function of a continuous internal opinion (in this case, intention), using Bayes’ theorem. Table 1 shows the equations to update the persons' intention every week. The level of intention in a given week (Eq. 7) depends on the behavior of those in the proximal network (Eq. 2) and perceived community (Eq. 3), the person’s behavior in the previous week (Eq. 4), current level of intention (Eq. 5), and the highest perceived utility amongst the LTPA sites in the person’s perception radius (Eq. 6).

**Table 1:** Equations to obtain change of intention per week

Eq. #	Equation	Terms
2	${p}_{i,t}=\frac{1}{n}\sum \limits_{x=1}^n{b}_{x,t-1}\left[\ln \left(\frac{\alpha_p}{1-{\alpha}_p}\right)\right]$	*p*_*i,t*_ is the increment or reduction of person *i*’s intention in week *t* due to the behavior of the proximal network, *n* is the number of people in the proximal network, *b*_*x,t-1*_ is the behavior of person *x* in the proximal network in week *t-1*, and *α*_*p*_ is the conditional likelihood that people in the proximal network will practice LTPA if it is the best option (*i.e.*, their act reflects the best thing to do)
3	${c}_{i,t}=\frac{1}{n}\sum \limits_{x=1}^n{b}_{x,t-1}\left[\ln \left(\frac{\alpha_c}{1-{\alpha}_c}\right)\right]$	*c*_*i,t*_ is the increment or reduction of person *i*’s intention in week *t* due to the behavior of the perceived community, *n* is the number of people in perceived community, *b*_*x,t-1*_ is the behavior of person *x* in the perceived community in week *t-1*, and *α*_*c*_ is the conditional likelihood that people in the perceived community will practice LTPA if it is the best option
4	${l}_{i,t}={b}_{i,t-1}\left[\ln \left(\frac{\alpha_b}{1-{\alpha}_b}\right)\right]$	*l*_*i,t*_ is the increment or reduction of person *i*’s intention in week *t* due to his previous behavior, *b*_*i,t-1*_ is the behavior of person *i* in week *t-1*, and *α*_*b*_ is the conditional probability that a person will practice LTPA if it is the best option
5	${w}_{i,t}=1-\frac{\left|{i}_{i,t-1}\right|}{\ln \left(\frac{0.97}{0.03}\right)}$	*w*_*i,t*_ represents the intention strength of person *i* in week *t*, *i*_*i,t-1*_ is the person *i*’s intention in week *t-1*
6	${y}_{h,i}=\left[\ln \left(\frac{u_{h,i}}{1-{u}_{h,i}}\right)\right]\frac{1}{r}$	*y*_*h,i*_ is the log-odds of the highest perceived utility (*u*_*h,i*_), *r* is a scaling factor (set by the modeler, 100 in this work) to re-scale the magnitude of the built environment’s influence
7	${i}_{i,t}={i}_{i,t-1}+{w}_{i,t}\left({p}_{i,t}+{c}_{i,t}+{l}_{i,t}+{y}_{h,i}\right)$	*i*_*i,t*_ is the level of intention of person *i* in week *t*

*LTPA* Leisure-time physical activity

In Eqs. 2, 3, 4, the log-odds of *α* can be interpreted as how confident someone is that the behavior of his neighbors (or his own behavior, in Eq. 4) represents the best option. Different values for *α* are possible, allowing for different weights of the person’s and neighbors’ behavior in affecting future levels of intention.

Lastly, level of intention in week *t* is transformed into the probability of practicing LTPA in that week by Eq. 8:

8 $$ Prob\left({b}_{i,t}\right)=\frac{e^{i,t}}{1+{e}^{i,t}} $$

In which *Prob(b*_*i,t*_*)* is the probability of person *i* practicing LTPA in week *t*, and *e*^*i,t*^ is the exponential of person *i*’s intention in week *t*. *Prob(b*_*i,t*_*)* is limited between 0.03 and 0.97 instead of 0 and 1 to account for the possibility that one could adopt the opposite behavior regardless of his current intention (for instance, being ill [for those with 0.97] or in vacation [for those with 0.03]). This probability is used to set each person’s behavior in week *t*. The entire cycle reinitiates in week *t + 1* beginning with Eq. 2.

### Model implementation

The model’s algorithm was implemented on NetLogo version 6.0.3 [33]. A verification protocol was followed to ensure the accuracy of the algorithm, consistency with the conceptual model, and avoid errors, omissions and bugs. The protocol included continuous reviewing of spelling, syntax and commands; monitoring of unexpected elements, actions, calculation, and outputs; and stress tests (i.e., using inputs and parameter values out the plausible range) [34, 35].

Spatial distribution of LTPA practice, population distribution of intention, and proportion of people practicing LTPA over time can be visualized using the model’s user interface and/or recorded for future analyses.

### Parametrization and calibration

We defined initial values and domains of each parameter in the model following the premises of pattern-oriented modeling [34], in which the patterns observed in real systems are used as a reference to obtain models that are more realistic in structure and, therefore, more useful and generalizable. Two population patterns were used as guidance for parametrization and calibration: temporal trends of LTPA (> 0 min/week) and population distribution of intention to practice LTPA.

Studies from around the world involving adult populations show temporal trends of LTPA are stable or increasing by very little, generally less than one percentage point a year, in periods varying from five to ten years (the period simulated in the model), with prevalence ranging from 35 to 50% [15–20].

Our search for temporal trends in the population distribution of intention to practice LTPA did not return any relevant information. We then searched for population-based cross-sectional studies and identified studies on population distributions of behavioral change stage — construct of the Transtheoretical Model [36] that is a *proxy* of intention. In general, distributions were U shaped, with higher proportions of people in the pre-contemplation (20–35%) and maintenance (25–45%) stages. Contemplation and preparation stages took turns as the third largest proportion (around 15% each), while the action stage presented the lowest values (around 5%) [37–40].

Therefore, the model was expected to reproduce scenarios in which LTPA was stable or presented slight increase over time, and a U-shaped distribution of intention. To monitor these patterns within the model, we extracted the proportion of people practicing LTPA and people with low (0.03 ≤ intention < 0.25), intermediate (0.25 ≤ intention ≤0.75), and high (0.75 < intention ≤0.97) intention.

After defining the expected population patterns for the model, we defined initial values and domains of parameters for which we found empirical data: size of proximal network [41–43] and perceived community [42, 44, 45], and amount/density of LTPA sites [46–48].

No empirical data was found on the size of persons’ perception radius. Therefore, this parameter was calibrated to match values reported in the literature for the size of perceived community [42, 44, 45] and amount/density of LTPA sites in persons’ neighborhoods [46–48]. Final value was set to 9.0 (domain = 0 to 50).

For the other parameters, we defined a plausible initial value and domain that would lead to the expected time trends of LTPA practice and the expected population levels of intention, since empirical data could not be found for parametrization. These parameters were calibrated simultaneously by trial and error, a procedure considered sufficient at the first stages to proceed with the analyses. In later stages, we identified the most relevant parameters and assessed sensitivity of outputs to input values.

Most of the empirical data used for parametrization and calibration came from urban settings within high-income Western countries. However, wide but plausible domains were set for all parameters to accommodate contexts not initially covered by the available data and allow thorough exploration of the parameters during sensitivity analyses.

Additional file 1: Table S1.2 displays all the parameters in the model and their domains and initial values.

### Consistency and sensitivity analysis

#### Consistency analysis

Consistency analysis was conducted to determine the number of replications required to reduce uncertainty in the outputs due to stochasticity (i.e., originated from random events) [49, 50].

Twenty sets were generated for each *m* number of replications (1, 5, 10, 20, 30, 40, 50, 60, 70, 80, 90, and 100). From every replication we extracted the proportion of people practicing LTPA and with low, intermediate, and high intention. Each set contained a distribution with *m* values for each output. Using the Vargha-Delaney A Test [51] we identified whether the 20 distributions including *m* replications were consistent against each other.

The Vargha-Delaney A Test is not a hypothesis testing procedure but still generates an effect size measure that takes values between 0 and 1, with a value of 0.5 indicating that the medians of two distributions with *m* replications are similar, showing a high consistency of results (i.e., less uncertainty due to stochasticity). Scores close to 0.44 or 0.56 indicate small differences, values around 0.36 or 0.64 express medium effect size, and scores lower than 0.29 or higher than 0.71 indicate large differences [50, 51].

For each *m* replications, median and maximum A score were calculated from the scores obtained comparing the first and the remaining 19 distributions generated. Line graphs were plotted to identify the minimum required number of replications that result in low uncertainty due to stochasticity.

#### Sensitivity analysis

We conducted individual and global sensitivity analyses. For individual sensitivity analyses, we used the parameter robustness technique, in which every parameter of interest is disturbed individually, while the remaining ones are kept in their initial values [49, 50]. Fourteen parameters (two related to personal attributes, four to social environment, and eight to built environment) were investigated (Additional file 2: Table S2.1). Temporal trends of LTPA and distribution of intention obtained from the scenarios with the new conditions were compared against the data obtained from the baseline scenario through the Vargha-Delaney A Test [51]. Scores close to 0.5 indicated that temporal trends were robust (i.e., not very sensitive) to alterations of the parameter’s values [50]. The farther the score is from 0.5, the more sensitive the model is to that particular parameter. Scores higher than 0.71 or lower than 0.29 indicate significant sensitivity [50, 51]. Line graphs and boxplots were used to provide summaries and visualize results.

For the global sensitivity analysis, selected parameters of interest were simultaneously disturbed [49]. We first selected the most influential parameters from the individual sensitivity analysis (Additional file 2: Table S2.2). Next, Latin Hypercube Sampling technique was employed to generated 100 scenarios by drawing values from each selected parameter to optimize the exploration and coverage of the whole domain of all parameters simultaneously [50]. Partial rank correlation coefficients and scatter plots were generated to analyze the correlation between each parameter and outputs that can be observed despite the simultaneous changes in all other parameters [49]. Results were summarized by minimum, maximum, mean, and standard-deviation. Effect sizes were interpreted as small (~ 0,2), average (~ 0,5) or high (~ 0,8) [52].

#### Outputs analyzed and statistical package

For all consistency and sensitivity analyses, we obtained and analyzed the proportion of people practicing LTPA and people with low, intermediate, and high intention at every 52 weeks in a total of 624 weeks, which is equivalent to yearly data for 12 years.

The statistical software R, version 3.2.2, and spartan package, version 2.3, were used to conduct the analyses.

### Availability of data and materials

The model code and user interface, R codes, original outputs of all replications, and full results generated for this work are available at 10.17605/OSF.IO/J2KAS. Parts of this information are also described in Additional files 1, 2, 3 and 4.

## Results

**Fig. 3:**
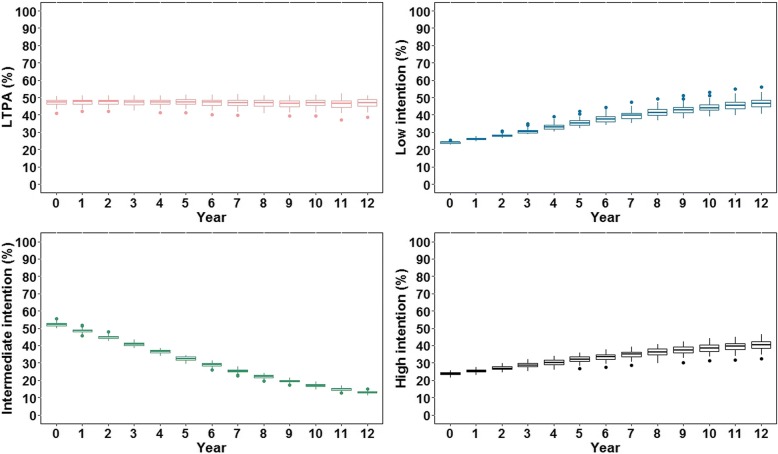
Outputs from the baseline scenario obtained at every 52 iterations (equivalent to yearly) and summarized from 80 replications. Outputs: red = proportion of people practicing leisure-time physical activity (LTPA); blue = proportion of people with low intention (0.03 ≤ intention < 0.25); green = proportion of people with intermediate intention (0.25 ≤ intention ≤0.75); black = proportion of people with high intention (0.75 < intention ≤0.97)

Figure 3 shows temporal trends of persons practicing LTPA and with low, intermediate and, high intention, generated from the scenario using the parameter values in Additional file 1: Table S1.2. This scenario was able to reproduce the temporal trends reported in the literature, showing a stable prevalence of LTPA of approximately 48% throughout the years, while the population distribution of intention exhibited a U shape, with increasing proportions of people with high and low intention over time.

### Consistency analysis

**Fig. 4:**
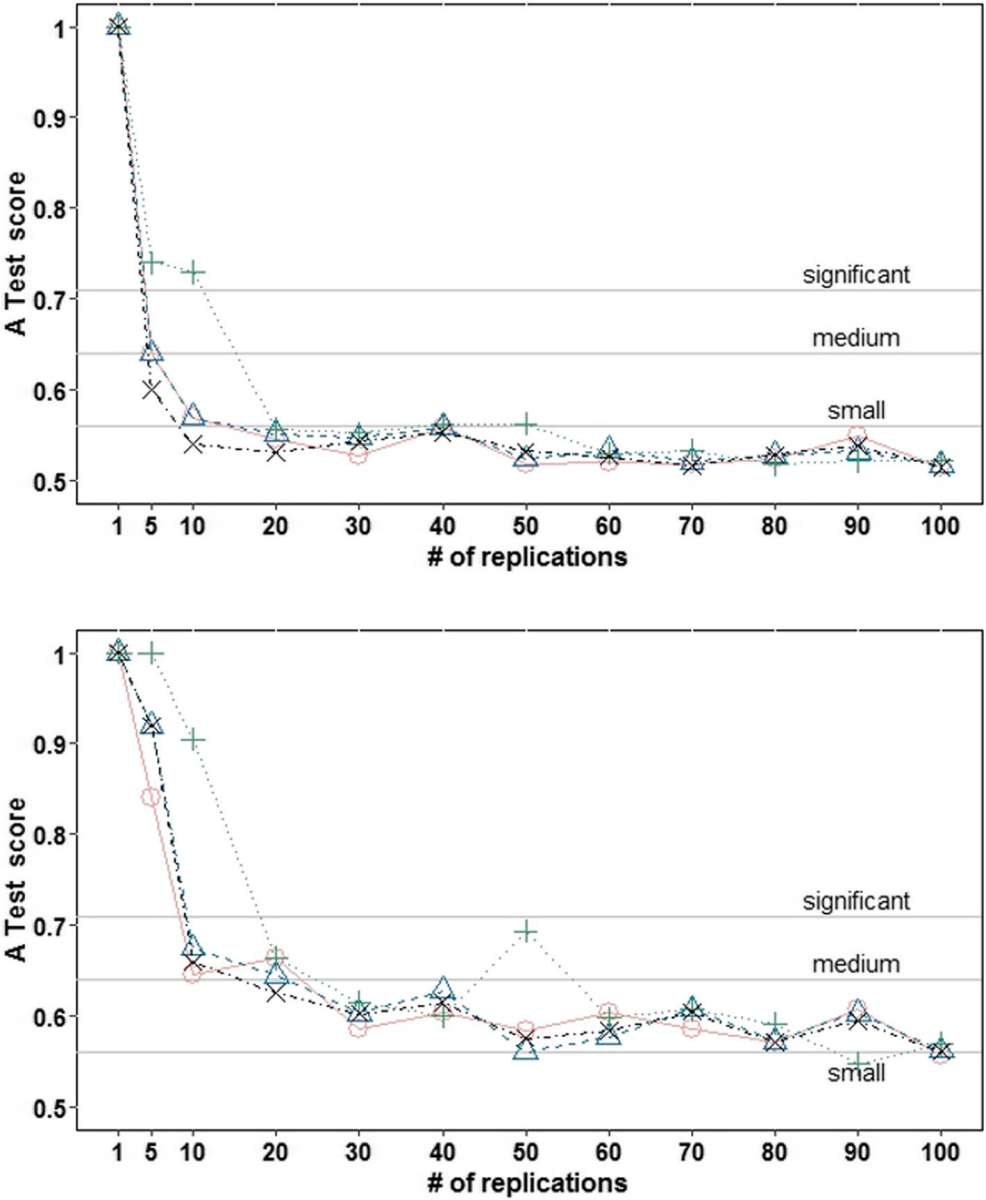
Median (upper chart) and maximum (lower chart) scores from the Vargha-Delaney A Test, for each number of replications, obtained at the 520th iteration (equivalent to the 10th year). Outputs: red = proportion of people practicing leisure-time physical activity; blue = proportion of people with low intention (0.03 ≤ intention < 0.25); green = proportion of people with intermediate intention (0.25 ≤ intention ≤0.75); black = proportion of people with high intention (0.75 < intention ≤0.97)

Results from the consistency analysis were summarized in 169 charts and 26 spreadsheets. Figure 4 displays the median and maximum scores from the Vargha-Delaney A Test for each number of replications tested, obtained at the 520th iteration (equivalent to the 10th year). Plots and spreadsheets of all other years (available at 10.17605/OSF.IO/J2KAS) have returned results similar to those portrayed in Fig. 4.

After 80 replications, the reduction of both the median and maximum A score became stable. Therefore, 80 replications were deemed as the minimum required to obtain time trends of LTPA practice and intention levels that are reliable and less influenced by stochasticity.

In all the other analyses conducted for this work, 80 replications were made for each scenario.

### Sensitivity analysis

Additional file 3 provides a summary of the observed results from the individual sensitivity analysis. From the 14 parameters tested, two had high influence on the proportion of people practicing LTPA: the size of the person’s perception radius, and the proportion of patches in the grid that are LTPA sites. Three parameters (the size of the perception radius, the proportion of patches that are LPTA sites, and the influence of the person’s behavior in the previous week over his current intention — *α*_*b*_ term in Eq. 4) had high influence on the proportion of people with low, intermediate, and high levels of intention.

Six parameters were selected for the global sensitivity analysis, two related to personal attributes (influence of the person’s behavior in the previous week over his current intention, size of the person’s perception radius), two to social environment (influence of the proximal network’s behavior over the person’s intention [*α*_*p*_ term in Eq. 2], influence of the perceived community’s behavior over the person’s intention [*α*_*c*_ term in Eq. 3]), and two to built environment (proportion of LTPA sites, mean quality score of LTPA sites) (Additional file 2: Table S2.2).

Table 2 displays a summary of the partial rank correlation coefficients, obtained from the global sensitivity analysis using the most influential parameters. Additional file 4, also generated from the global sensitivity analysis, exemplifies scatter plots obtained at the 520th iteration (equivalent to the 10th year) assessed in order to identify non-linear effects.

**Table 2:** Partial rank correlation coefficients obtained from the global sensitivity analysis

Parameters	% LTPA				% Low intention				% Intermediate intention				% High intention			
	min	max	$\overline{x}$	sd	min	max	$\overline{x}$	sd	min	max	$\overline{x}$	sd	min	max	$\overline{x}$	sd
Individual attributes																
alpha.behavior	0.18	0.36	0.31	0.06	0.61	0.80	0.70	0.06	− 0.95	− 0.89	− 0.91	0.02	0.63	0.69	0.68	0.02
perception.radius	0.92	0.94	0.93	0.01	−0.84	− 0.80	− 0.83	0.01	− 0.10	0.74	0.65	0.24	0.81	0.86	0.84	0.02
Social environment																
alpha.network	−0.16	−0.07	−0.11	0.03	0.23	0.28	0.26	0.02	−0.46	−0.28	− 0.37	0.05	− 0.11	−0.04	− 0.06	0.02
alpha.comm	−0.07	0.01	−0.05	0.02	0.19	0.22	0.21	0.01	−0.29	−0.08	− 0.15	0.06	− 0.06	−0.02	− 0.04	0.01
Built environment																
prop.ltpa.sites	0.69	0.76	0.72	0.02	−0.52	−0.43	− 0.47	0.03	0.10	0.30	0.25	0.05	0.48	0.55	0.51	0.02
mean.ql	0.18	0.24	0.21	0.02	−0.17	−0.04	− 0.11	0.04	0.03	0.26	0.07	0.07	0.10	0.13	0.12	0.01

Coefficients assume values ranging from −1 (perfect negative correlation) to + 1 (perfect positive correlation), where 0 means no correlation

*%* proportion of [*output*], *LTPA* leisure-time physical activity, *sd* standard-deviation

*alpha.behavior* influence of a person’s behavior in the previous week over his current intention

*perception.radius* size of the person’s perception radius

*alpha.network* influence of the proximal network’s behavior over the person’s intention

*alpha.comm* influence of the perceived community’s behavior over the person’s intention

*prop.ltpa.sites* proportion of LTPA sites

*mean.ql* mean quality score of LTPA sites

Global sensitivity analyses indicated that the model is highly sensitive to three parameters: the influence of the person’s behavior in the previous week over his current intention, the size of the person’s perception radius, and the proportion of patches in the grid that are LTPA sites (Table 2).

The influence of the person’s behavior in the previous week over his current intention had a high impact on the proportion of people in each level of intention. The stronger the influence of the previous behavior over current intention, the more persons presented high or low intention, while fewer persons remained in the intermediate range. However, the influence over the proportion of people practicing LTPA was small (Table 2).

The size of a person’s perception radius and the proportion of LTPA sites had a high impact on temporal trends of LTPA practice and a moderate to high effect on intention levels. The wider the perception radius and the higher the proportion of LTPA sites, the higher the proportion of people practicing LTPA and with intermediate and high intention, and the lower the proportion of people with low intention (Table 2). However, the relationship was non-linear. Small increments when the radius is narrower led to greater changes in the proportion of people practicing LTPA and in each level of intention (Additional file 4: Figure S4.2). As for the proportion of LTPA sites, changes in the proportion of people practicing LTPA and in each level of intention were observed only at the extremities of the values domain (Additional file 4: Figure S4.5).

The influence of the proximal network’s behavior over the person’s intention had a low impact on the proportion of people practicing LTPA and on the proportion with high intention, whereas an average impact on the proportion of those with low and intermediate intention. The influence of the perceived community’s behavior over a person’s intention had a low impact on the four proportions. For both parameters, the stronger the effect of the social environment, the higher the proportion of people with low intention, and the lower the proportion of those with intermediate intention, an unexpected result (Table 2).

The model displayed low sensitivity to the mean quality score of LTPA sites. As the quality increased, the proportion of people practicing LTPA and with high intention increased only slightly (Table 2).

## Discussion

Our goal was to develop an agent-based model that can enable researchers to explore the emergence and evolution of population patterns of LTPA among adults, emerging from the interaction between the individuals’ psychological attributes and the built and social environments in which they live. Analyses conducted with the model have shown that time trends of LTPA practice and population distribution of levels of intention are similar those reported in literature [15–22, 37–40], which suggests the model was capable of capturing the behavior of the emulated phenomenon. Our analyses also identified the elements and mechanisms that significantly influence the temporal trends in the model, particularly the influence of the person’s behavior in the previous week over his current intention, the size of the person’s perception radius, and the proportion of patches in the grid that are LTPA sites.

The model was based on a conceptual model designed from a literature review and expert assessment [30] in order to maintain coherence and consistency with the best evidence available in the field. It is strongly based on the premises of systems thinking, expressly suggesting the dynamic mechanisms and relationships between individual psychological attributes and social and built environments. In our model, psychological attributes are considered the strongest proximal determinants of LTPA, a relationship that is dynamically moderated by the built environment — moderation that depends on the person’s psychological attributes — and influenced by both the social environment and the behavior itself. The literature review, expert opinions [30], and the model’s initial results suggest that this is a plausible representation of reality.

Three elements and mechanisms exhibited stronger influence on time trends of people practicing LTPA and levels of intention: the influence of the person’s behavior in the previous week over his current intention, size of the person’s perception radius, and proportion of LTPA sites in the model. Other three elements and mechanisms had lower effect: proximal network’s and perceived community’s behaviors influence on the person’s intention, and mean quality score of LTPA sites. These six parameters — particularly the first three — should receive special attention when calibrating the model for future works.

Individual and global sensitivity analyses shown some unexpected results and non-linear dynamics. One example is the effect of the social environment. The influence of the proximal network’s and perceived community’s behavior over the person’s intention presented small effect sizes, but in an undesired way: the stronger the social influence, the higher the proportion of people with low intention to practice LTPA. This may have happened because, on average, persons had more contact with others who did not practice LTPA, as the prevalence of LTPA was usually less than 50% for the investigated scenarios. This situation is, however, common to several locations [15, 16, 18–22] and should be considered when planning campaigns and initiatives promoting LTPA using the social environment as one of the elements.

Another example is the positive effect of increasing the proportion of LTPA sites over the temporal trends of people practicing LTPA and levels of intention. This positive effect can be split into two distinct phases. When a certain proportion of sites is achieved, additional positive effects on LTPA practice may depend on a much stronger and sustained investment on infrastructure to translate it into significant changes on LTPA practice. Indeed, at least one meta-analysis [53], four systematic reviews of quantitative studies [54–57] and one of qualitative studies [58] shown positive associations between LTPA practice and distance to or density of LTPA sites. However, a possible non-linear relationship between density of LTPA sites and LTPA prevalence is still to be investigated.

The most important contribution of our agent-based model is the systems perspective it provides on the issue, being more focused on the system’s structure [59, 60]. As a general model, the usefulness of this model to LTPA promotion does not hinge on its use as a tool for evaluation of policy interventions in a particular location, but as an additional means to obtain deeper comprehension on the factors influencing population patterns of LTPA and how they interact shaping and sustaining the observed patterns. It is noteworthy, however, that the model can, in principle, be extended and validated to represent real locations and test policy options specific to these places.

There is space for improvements in future versions of the model, especially with the advent of more evidence that can inform the model structure and parameters. Future versions could:

- Include perception and decision capacities to LTPA sites, allowing them to adapt to the characteristics of people in their surroundings;
- Allow certain parameters to vary among persons, such as perception radius or the relative weights given to quality, access, and availability of favorite activity when raking LTPA sites;
- Break down intention into its psychological precedents, such as attitude and self-efficacy;
- Consider attributes of LTPA sites, for example, financial cost of use and distance, separately.

However, the current version of the model is already capable of reproducing population time trends of LTPA and distributions of intention observed in real settings and can be used to explore some of the dynamic mechanisms generating them.

## Conclusion

The agent-based model developed in this work is appropriate to investigate the emergence and evolution of population patterns of LTPA among adults, emerging from the interaction between the individuals’ psychological attributes and the built and social environments in which they live. Initial results showed that three elements and mechanisms displayed stronger influence on time trends of people practicing LTPA and levels of intention within the model: the influence of the person’s behavior in the previous week over his current intention, size of the person’s perception radius, and proportion of patches in the model that are LTPA sites.

Asides from improving the model, future work includes testing scenarios and interventions — either hypothetical or equivalent to real situations — to gain a better understanding of the conditions that generate either observed or desired population patterns. The model is available to download (10.17605/OSF.IO/J2KAS) and can be used, adapted, and improved by others for this purpose. We expect that work deriving from our model can inform researchers and policymakers on the design of more effective population-level physical activity interventions.

Population-based initiatives will probably continue to present results below the desired level until we seek to understand the dynamics and structures that support population patterns of LTPA. The results achieved by this work show that it is possible to approach population patterns of LTPA in a way that is more in line with a systems science perspective and with the idea that physical activity is a complex, multidimensional and multidetermined behavior.

## Additional files

Additional file 1: Full description of the agent-based model. (PDF 321 kb)

Additional file 2: Parameters and values investigated in sensitivity analyses. (PDF 85 kb)

Additional file 3: Summary of results of individual sensitivity analysis. (PDF 113 kb)

Additional file 4: Results of global sensitivity analysis (year 10). (PDF 712 kb)

## Abbreviations

LTPA: Leisure-time physical activity
