# Supplementary material for: Exploring the emergence and evolution of population patterns of leisure-time physical activity through agent-based modelling
Source: Int J Behav Nutr Phys Act. 2018 Nov 19;15:112. doi: 10.1186/s12966-018-0750-9 (PMC6245872; doi:10.1186/s12966-018-0750-9)
Supplement: Supplementary file 1 — Full description of the agent-based model. (PDF 321 kb) [file 12966_2018_750_MOESM1_ESM.pdf]

Additional file 1 of "Exploring the emergence and evolution of population patterns of leisure-time physical activity through agent-based modelling", by Leandro M. T. Garcia, Ana V. Diez Roux, André C. R. Martins, Yong Yang, and Alex A. Florindo.

FULL DESCRIPTION OF THE AGENT-BASED MODEL

This document was organized according to the Overview, Design Concepts, and Details + Decision protocol[1].

OVERVIEW

Purpose

The model has been developed to investigate population patterns of LTPA in adults resulting from the interaction between the individuals' psychological attributes and the attributes of the built and social environments in which they live.

Entities, state variables, and scales

The model has two types of agent: persons and LTPA sites. The attributes that characterize each type of agent are presented in Table S1.1. Apart from the attributes therein described, each person keeps in his memory a list of LTPA sites within his perception radius (a limited area within the grid that the agent can observe), the perceived utility of these sites, and the people who compose his proximal network.

The model does not emulate any particular environment or community. The physical space is represented by a square grid with non-contiguous sides. Patches are characterized as being or not a LTPA site. Only patches characterized as non-LTPA sites contain persons, as the distance between persons and sites must be higher than zero.

The dimensions are not equivalent to any real length or area. The default grid dimensions (50 x 50 patches) were defined alongside the population size (2,000), so that more relevant

factors, such as the number of people and LTPA sites within persons' perception radius, were similar to those reported by the literature, taking also computational costs to run the model into consideration.

Time is discrete, and each iteration is equivalent to one week, a timeframe frequently used to investigate physical activity behavior. We assessed a period of 10 years (520 iterations).

Table S1.1. Attributes of persons and LTPA sites.

**Attribute**	**Scale and domain**
Persons	
Location	Continuous, Cartesian coordinates from 0 to 49 in both axes
Intention	Continuous, from 0.03 to 0.97
Favorite LTPA	Discrete, one or none among 10 LTPAs available in the model
Behavior	Binary, -1 (no LTPA in the week) and +1 (LTPA in the week)
LTPA sites	
Location	Discrete, Cartesian coordinates from 0 to 49 in both axes
Quality	Continuous, from 0 to 1
Number and type of LTPAs available	List containing *n* of 10 LTPAs available in the model

LTPA: leisure-time physical activity.

Process overview and scheduling

Every week, each person's intention is updated based on his behavior and the behavior of his proximal network and perceived community in the previous week. Then, each person decides whether he will practice LTPA during the current week, based on the new level of intention and conditional to the perceived built environment. The entire process is synchronic (*i.e.*, every person updates the same attributes at the same time) and happens in a weekly basis.

DESIGN CONCEPTS

Theoretical and empirical background

The agent-based model is grounded on the conceptual model developed by Garcia et al.[2].

Individual decision-making

Every week, each person decides whether he will practice LTPA or not. The person's decision-making process is based on the assumptions of limited rationality (*i.e.*, persons do not have complete and perfect information to make decisions)[3], inductive thinking[4], social practice[5,6], and opinion dynamics[7,8].

There are no explicit goals or consequences underpinning the decision-making process. However, persons tend to maintain their habitual behavior, while seeking to adapt it according to the behavior observed from their proximal network and community. The decision-making process occurs weekly on a probabilistic basis, based on the person's intention to practice LTPA conditioned to his perception of the built environment.

The observation of the proximal network and community behaviors modifies the person's intention, therefore impacting the decision-making process.

Time affects the decision-making process strengthening intention, either towards to practice LTPA or not. The stronger the intention, the lesser the influence of other factors (*e.g.*, built and social environments) over the decision-making process.

LTPA sites have no decision-making process.

Learning

Agents do not change their decision-making rules over time, so the model does not include learning processes.

Individual sensing

Each person is capable of observing the behavior of two groups of people:

a) Proximal network: comprised by people with whom he has close relationships (such as friends and relatives);
b) Perceived community: group of people within the person's perception radius.

Each person infers the social norms of the entire community looking at the behavior of the perceived community. Only the behavior of a fraction of the perceived community is observed every week to reflect the fact that no person has full and perfect knowledge about the behavior of the entire community, nor even of the people within his perception radius.

Each person also perceives the LTPA sites within his perception radius. The utility attributed by each person to each site depends on the site's attributes and subjective factors influencing the person's assessment.

The mechanisms by which persons obtain information and the cost to get and process them are not part of the model.

LTPA sites have no perceptive capacity, so they are not sensible nor react to what occurs during the simulations.

Individual prediction

Persons do not possess predictive capacity, so they cannot predict possible consequences of their actions nor consider this information when making decisions.

Interaction and collectives

Each person interacts directly with his proximal network and perceived community. At the same time, he can be part of other persons' proximal network and perceived community. The interaction occurs through the perception of the behavior adopted in the previous week by other people.

The proximal network is formed selecting the *k* closest persons in the grid. Each link has a probability *p* to be exchanged for a link with any other person outside the initial proximal network. This procedure aims to ensure that the proximal network is mostly formed by people with similar social and environmental influences. At the same time, it allows the formation of a small-world network[9] and increases the variability of environmental influences.

The perceived community is formed by people within the person's perception radius. Every week, the behavior of only a fraction of the perceived community is accessible to the person (more details in the "Individual sensing" subsection).

Heterogeneity

Agents of the same type have identical set of attributes and decision-making processes. Only the values given to each attribute change.

Stochasticity

The following elements are defined stochastically when setting the model up:

a) LTPA sites attributes: location, quality, number and type of LTPAs available;
b) Persons' attributes: location, initial intention and behavior, and favorite LTPA;
c) Person–LTPA-site dyad: scaling factor that represents the person's subjective assessment of a given site;
d) Exchange of links of the initial proximal network.

Two stochastic processes occur throughout the simulation, every week:

a) Persons' decision about practicing LTPA;
b) Definition of a subset of people within the perceived community whose behavior will be observed by the person.

Observation

Modelers can observe the spatial distribution of LTPA practice, the population distribution of intention, and the proportion of people practicing LTPA in the whole population and by subgroups. It is possible to follow temporal trends and obtain data from any week.

DETAILS

Implementation details

The most updated version and documentation of the model can be accessed at https://doi.org/10.17605/OSF.IO/J2KAS.

Initialization

A proportion of patches, defined by the parameter *prop.ltpa.sites* (further details about all parameters can be seen in Table S1.2, at the end of this document), is designated as LTPA sites and randomly positioned in the grid. Each site gets a quality score, drawn from a normal distribution with mean and standard deviation defined by parameters *mean.ql* and *sd.ql*, respectively. Next, the number of LTPAs available in each site is drawn from a uniform distribution, delimited by the parameters *min.activities* and *max.activities*. Finally, the types of LTPAs available in each site are defined, drawing from a list of 10 possible activities using a uniform distribution (10 values, 1 to 10) — 10 activities give enough variability of LTPAs available in the sites and of favorite activities among people.

Persons are placed randomly in the grid over patches that do not represent LTPA sites. Every person sets, randomly, none or one of the 10 available LTPAs as his favorite, drawing from a uniform distribution (11 values, 0 to 10, in which 0 represents not enjoying any LTPA). Next, each person incorporates in his memory a list of LTPA sites within his perception radius. Every person extracts and stores the following attributes from each of these sites:

a) Quality score;

b) Accessibility score, which is the Euclidian distance between the person and the site. The distance represents how easily the person can reach the site, including factors such as traffic, safety, physical proximity, cost and ease of transportation to it[2]. The longer the distance, the worse the access;

c) Whether his favorite LTPA is available at the site.

A scaling factor, equivalent to the person's subjective assessment, is set to every person–LTPA-site dyad. The value comes from a normal distribution with mean and standard deviation defined by parameters *mean.perception* and *sd.perception*, respectively. These four pieces of information are used to calculate the perceived utility of each site, also stored in the person's memory (see Equation 1, in the "Submodels" subsection).

Every person is assigned a level of intention, which is defined in two steps:

a) A proportion of people, defined by parameter *first.prop*, receives an intention value drawn from a uniform distribution, delimited by parameters *first.min.in* and *first.max.in*;

b) The remaining receive an intention value taken from another uniform distribution, delimited by parameters *second.min.in* and *second.max.in*.

These two steps allow for more flexibility when obtaining the population distribution of intention. Persons who do not enjoy LTPA have their intention defined as the minimum possible value.

Each person also receives a behavior (*i.e.*, did or did not practice LTPA in the week), with probability equal to his intention. Persons who do not have any LTPA site within their perception radius are assigned to not having practiced the behavior in the past week.

Each person's proximal network is defined through the procedure described in the "Interaction and collectives" subsection. The network size is defined by the *network.size* parameter. The probability of a link of the initial proximal network be exchanged for a link with

someone outside of it is defined by *rewiring.probab*. The perception radius size is defined by the *perception.radius* parameter.

Initial configurations of the same scenario may differ between replications as they mostly rely on stochastic processes. Figure S1.1 exemplifies a grid at the end of the initialization process.

Input data

There are no external sources, such as databases or outputs from other models, used as inputs for the model initialization or other processes throughout the simulations.

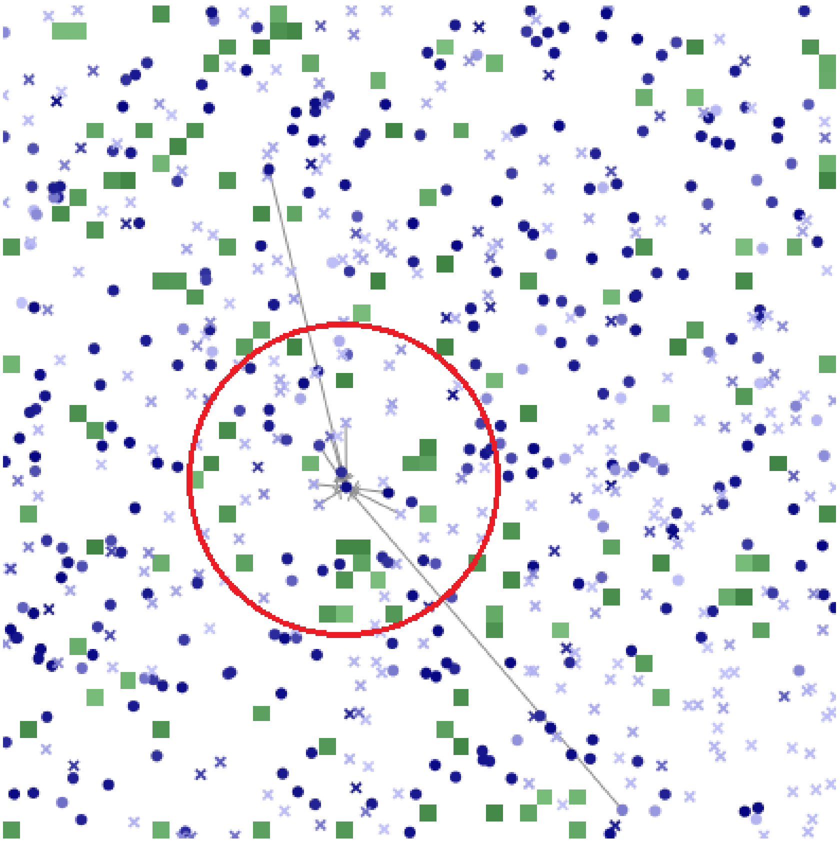

Figure S1.1. Example of a grid after initialization. Green patches are leisure-time physical activity (LTPA) sites. The darker the green, the higher the sites' quality. Persons are indicated by crosses (did not practice LTPA in the previous week) and circles (practiced LTPA in the previous week). The darker the blue, the higher the person's intention. Gray lines represent the person's proximal network and the red circle his perception radius.

Submodels

At the initialization, every person stores in his memory the perceived utility of each LTPA site within his perception radius. The perceived utility is obtained through Equation 1:

$$u_{s,i} = v_{i,s}\left(\frac{q_s}{3} + \frac{1}{3z_{i,s}} + \frac{m_{i,s}}{3}\right) \quad (1)$$

In which $u_{s,i}$ is the utility of site s as perceived by person i, $v_{i,s}$ is the subjective assessment (scaling factor) given by person i to site s, q_s is the quality of site s, $z_{i,s}$ is the access of person i to site s, and $m_{i,s}$ shows whether the site s offers the favorite LTPA of person i.

These three attributes (quality, access, and availability of favorite activity) have the same weight in the perceived utility as evidence about the relative weight people give to these attributes is lacking. The only attribute obtained directly from the sites is quality (q_s), which ranges from 0 to 1. Access ($z_{i,s}$) and available activities ($m_{i,s}$) depend on the information of each person–LTPA-site dyad. Access ($z_{i,s}$) is represented in the model by the Euclidian distance between person and site (*i.e.*, the shorter the distance, the better the access). The term $m_{i,s}$ can take two values: 0 (the person's favorite LTPA is not offered at the site) or 1 (the person's favorite LTPA is offered at the site).

Instead of ranging from 0 to 1, $u_{s,i}$ ranges from 0.03 (as bad as the site can be, it is still better than having none) to 0.97 (as good as the site can be, there is always a cost to reach or use it). Each person also stores in his memory the site with the highest perceived utility and its value ($u_{h,i}$).

Every week, the person's intention is updated based on his behavior in the previous week, as well as the past behavior of those who are in his proximal network and perceived community. This updating process is based on an extension of the Continuous Opinions and Discrete Actions (CODA) model[8]. The model assumes that some discrete actions (in this case, practicing LTPA or not) are a function of a continuous internal opinion (in this case, intention). In the CODA model, a person's opinion is updated observing the neighbor's actions, using Bayes'

theorem. Martins' model[8] has been extended to this work to obtain the influence of the proximal network and perceived community through Equations 2 and 3, respectively:

$$p_{i,t} = \frac{1}{n}\sum_{x=1}^{n} b_{x,t-1} \, [\ln\left(\frac{\alpha_p}{1-\alpha_p}\right)] \quad (2)$$

$$c_{i,t} = \frac{1}{n}\sum_{x=1}^{n} b_{x,t-1} \, [\ln\left(\frac{\alpha_c}{1-\alpha_c}\right)] \quad (3)$$

In which $p_{i,t}$ ($c_{i,t}$) is the increment or reduction of person *i*'s intention in week *t* due to the behavior of the proximal network (perceived community), *n* is the number of people in the proximal network (perceived community), $b_{x,t-1}$ is the behavior of person *x* in the proximal network (perceived community) in week *t-1*, and α_p (α_c) is the conditional likelihood that people in the proximal network (perceived community) will practice LTPA if it is the best option.

In Equations 2 and 3, $b_{x,t-1}$ can have two values: +1 (practiced LTPA in the previous week) or -1 (did not practiced). Therefore, if all people within the proximal network or perceived community practiced LTPA in the previous week, the result is +1. The result will be -1 if none of them practiced it, and 0 if half of them did. This means that the magnitude of the increment or reduction of intention due to the social environment depends on how prevailing the behavior is within the proximal network and perceived community.

In Equation 2, *n*, defined by the parameter *network.size*, is identical for all persons, and for each person contains the same set of people every week. In Equation 3, *n* depends on how many people are within the person's perception radius, which size is defined by *perception.radius*, and the proportion of people observed every week, defined by *observed.comm*. That means that every person has his own *n*, which can refer to a different set of people every week.

The second part of Equations 2 and 3 calculates the log-odds of the conditional probability that people within the proximal network or perceived community will practice LTPA if it is the best option. It can be interpreted as how confident someone is that the behavior of their

neighbors represents the best option (*i.e.*, their act reflects the best thing to do). Equations 2 and 3 allow for different α values, enabling different weights to the behavior of the proximal network and the perceived community. In case there is no one in the perceived community, $c_{i,t}$ is zero.

Both $p_{i,t}$ and $c_{i,t}$ are expressed in log-odds. The use of log-odds in these and other equations cancels the normalization constant from the Bayes' theorem, simplifying arithmetic operations[8].

Apart from the social environment, the person's behavior in the previous week also influences his current level of intention. Equation 4 calculates this influence, also from an adaptation of the Martins' model[8]:

$$l_{i,t} = b_{i,t-1}[\ln\left(\frac{\alpha_b}{1-\ \alpha_b}\right)] \tag{4}$$

In which $l_{i,t}$ is the increment or reduction of person i's intention in week t due to his previous behavior, $b_{i,t-1}$ is the behavior of person i in week t-1, and α_b is the conditional probability that a person will practice LTPA if it is the best option. As in Equations 2 and 3, the term $b_{i,t-1}$ may take two values (+1 or -1), the second part calculates the log-odds of the conditional probability that a person will practice LTPA if it is the best option, and $l_{i,t}$ is expressed in log-odds.

Every week, a new level of intention is calculated from Equation 7, using as inputs the results of Equations 2 to 6:

$$w_{i,t} = 1 - \frac{|i_{i,t-1}|}{\ln\left(\frac{0.97}{0.03}\right)} \tag{5}$$

$$y_{h,i} = [\ln\left(\frac{u_{h,i}}{1-u_{h,i}}\right)]\frac{1}{r} \tag{6}$$

$$i_{i,t} = i_{i,t-1} + w_{i,t}(p_{i,t} + c_{i,t} + l_{i,t} + y_{h,i}) \tag{7}$$

In which $w_{i,t}$ represents the intention strength of person *i* in week *t*, $i_{i,t-1}$ is the person *i*'s intention in week *t-1*, $y_{h,i}$ is the log-odds of the highest perceived utility ($u_{h,i}$), and *r* is a scaling factor to re-scale the magnitude of the built environment's influence. Parameters $i_{i,t}$, $i_{i,t-1}$, $p_{i,t}$, $c_{i,t}$, $l_{i,t}$, and $y_{h,i}$ are expressed in log-odds.

Equation 5 ensures that the closer the intention is from its extreme values (*i.e.*, 0.03 or 0.97), the lesser the built and social environments and the behavior on the previous week influence changes in intention. It accounts for the fact that people with strong intentions are less affected by environmental cues[2].

In Equation 6, the term $u_{h,i}$ is transformed into log-odds for two reasons. First to keep the same scale as the other terms' in Equation 7. Second, in the original scale, an $u_{h,i}$ equals to 0.5 represents an environment that does not affect people's behavior (log-odds equal to zero). Values over 0.5 (*i.e.*, better environments) change intention in a positive way, while lower values (*i.e.*, worst environments) have a negative influence.

Finally, Equation 8 transforms intention in week *t* into the probability of practicing LTPA in the same week:

$$Prob(b_{i,t}) = \frac{e^{i,t}}{1 + e^{i,t}} \qquad (8)$$

In which $Prob(b_{i,t})$ is the probability of person *i* practicing LTPA in week *t*, and $e^{i,t}$ is the exponential of person *i*'s intention in week *t*. Equation 8 transforms the previous log-odds values back to the original scale. $Prob(b_{i,t})$ is limited between 0.03 and 0.97 instead of 0 and 1, to account for the possibility that a person could adopt the opposite behavior regardless of his current intention . In case there is no LTPA site within the person's perception radius, the probability of practice is 0. This probability is used to set each person's behavior in week *t*.

The entire cycle reinitiates in week *t+1* beginning with Equation 2.

Table S1.2 summarizes all parameters used to define a scenario and from which the equations' terms are generated.

Table S1.2. Model's parameters.

Parameter	Meaning	Scale and domain	Initial value
Population			
first.prop	Proportion of people in first range of intention distribution at the start of the simulation	Continuous, 0 to 1	0.25
first.min.in	Lower bound of the first range of intention distribution at the start of the simulation	Continuous, 0.03 to 0.97	0.03
first.max.in	Upper bound of the first range of intention distribution at the start of the simulation	Continuous, 0.03 to 0.97	0.30
second.min.in	Lower bound of the second range of intention distribution at the start of the simulation	Continuous, 0.03 to 0.97	0.31
second.max.in	Upper bound of the second range of intention distribution at the start of the simulation	Continuous, 0.03 to 0.97	0.97
α_b (alpha.behavior)	Influence of the person's behavior in the previous week over his current intention	Continuous, 0 to 1	0.5014
perception.radius	Size of the person's perception radius	Continuous, 0 to 50	9.0
Social environment			
network.size	Size of the proximal network	Discrete, 1 to 1,999	10
rewiring.probab	Probability that a link within the initial proximal network will be exchanged by a link with a person outside the network	Continuous, 0 to 1	0.15
observed.comm	Proportion of people within the perception radius observed every week	Continuous, 0 to 1	0.25
α_p (alpha.network)	Influence of the proximal network's behavior over the person's intention	Continuous, 0 to 1	0.5012
α_c (alpha.comm)	Influence of the perceived community's behavior over the person's intention	Continuous, 0 to 1	0.5010

Table S1.2. Model's parameters (*continuation*).

Parameter	Meaning	Scale and domain	Initial value
Built environment			
prop.ltpa.sites	Proportion of LTPA sites	Continuous, 0 to 1	0.015
min.activitites	Minimum number of activities available in LTPA sites	Discrete, 1 to 10	1
max.activities	Maximum number of activities available in LTPA sites	Discrete, 1 to 10	10
mean.ql	Mean quality score of LTPA sites	Continuous, 0 to 1	0.5
sd.ql	Standard deviation of quality score of LTPA sites	Continuous, 0 to 1	0.15
mean.perception	Mean of scaling factor representing the persons' subjective assessment of LTPA sites	Continuous, 0 to +∞	1.0
sd.perception	Standard deviation of scaling factor representing the persons' subjective assessment of LTPA sites	Continuous, 0 to +∞	0.15
r	Scaling factor of the built environment's influence	Continuous, 1 to +∞	100

LTPA: leisure-time physical activity.

REFERENCES

1. Müller B, Bohn F, Dreßler G, Groeneveld J, Klassert C, Martin R, et al. Describing human decisions in agent-based models–ODD+ D, an extension of the ODD protocol. Environmental Modelling & Software. 2013;48:37-48.

2. Garcia LMT, Diez Roux AV, Martins ACR, Yang Y, Florindo AA. Development of a dynamic framework to explain population patterns of leisure-time physical activity through agent-based modeling. International Journal of Behavioral Nutrition and Physical Activity. 2017;14:111.

3. Simon HA. Theories of bounded rationality. In: McGuire CB, Radner R, editors. Decision and organization. Amsterdam: North-Holland Publishing Company; 1972.

4. Arthur WB. Inductive reasoning and bounded rationality. American Economic Review. 1994;84(2):406-11.

5. Nettleton S, Green J. Thinking about changing mobility practices: how a social practice approach can help. Sociology of Health & Illness. 2014;36(2):239-51.

6. Rutten A, Gelius P. The interplay of structure and agency in health promotion: integrating a concept of structural change and the policy dimension into a multi-level model and applying it to health promotion principles and practice. Social Science & Medicine. 2011;73(7):953-9.

7. Acemoglu D, Ozdaglar A. Opinion dynamics and learning in social networks. Dynamic Games and Applications. 2011;1(1):3-49.

8. Martins ACR. Continuous opinions and discrete actions in opinion dynamics problems. International Journal of Modern Physics C. 2008;19(4):617-24.

9. Watts DJ, Strogatz SH. Collective dynamics of 'small-world' networks. Nature. 1998;393(6684):440-2.
