# Supplementary material for: Exploring the emergence and evolution of population patterns of leisure-time physical activity through agent-based modelling
Source: Int J Behav Nutr Phys Act. 2018 Nov 19;15:112. doi: 10.1186/s12966-018-0750-9 (PMC6245872; doi:10.1186/s12966-018-0750-9)
Supplement: Supplementary file 2 — Parameters and values investigated in sensitivity analyses. (PDF 85 kb) [file 12966_2018_750_MOESM2_ESM.pdf]

Additional file 2 of "Exploring the emergence and evolution of population patterns of leisure-time physical activity through agent-based modelling", by Leandro M. T. Garcia, Ana V. Diez Roux, André C. R. Martins, Yong Yang, and Alex A. Florindo.

PARAMETERS AND VALUES INVESTIGATED IN SENSITIVITY ANALYSES

Table S2.1. Parameters and domains investigated in the **individual** sensitivity analysis.

**Parameters**	**Domain**		
	**Lower bound**	**Upper bound**	**Increment**
Population			
Influence of the person's behavior in the previous week over his current intention (alpha.behavior)	0.5000	0.5020	0.0002
Size of the person's perception radius (perception.radius)	5	13	1
Social environment			
Size of the proximal network (network.size)	2	20	2
Proportion of people within the perception radius observed every week (observed.comm)	0.10 – 0.25 – 0.33 – 0.50 – 0.66 – 0.75 – 0.90		
Influence of the proximal network's behavior over the person's intention (alpha.network)	0.5000	0.5020	0.0002
Influence of the perceived community's behavior over the person's intention (alpha.comm)	0.5000	0.5020	0.0002

Table S2.1. Parameters and domains investigated in the **individual** sensitivity analysis (*continuation*).

Parameters	Domain		
	**Lower limit**	**Upper limit**	**Increment**
Built environment			
Proportion of LTPA sites (prop.ltpa.sites)	0.005	0.05	0.005
Minimum number of activities available in LTPA sites (min.activities)	1	10	1
Maximum number of activities available in LTPA sites (max.activities)	1	10	1
Mean quality score of LTPA sites (mean.ql)	0	1	0.1
Standard deviation of quality score of LTPA sites (sd.ql)	0.00	0.50	0.05
Mean of scaling factor representing the persons' subjective assessment of LTPA sites (mean.perception)	0.6	1.4	0.1
Standard deviation of scaling factor that represents the people's subjective assessment about the LTPA sites (sd.perception)	0.00	0.50	0.05
Standard deviation of scaling factor representing the persons' subjective assessment of LTPA sites (r)	20	200	20

LTPA: leisure-time physical activity.

Table S2.2. Parameters and domains investigated in the **global** sensitivity analysis.

**Parameters**	**Domain**	
	**Lower bound**	**Upper bound**
Population		
Influence of the person's behavior in the previous week over his current intention (alpha.behavior)	0.500	0.503
Size of the person's perception radius (perception.radius)	1	15
Social environment		
Influence of the proximal network's behavior over the person's intention (alpha.network)	0.500	0.503
Influence of the perceived community's behavior over the person's intention (alpha.comm)	0.500	0.503
Built environment		
Proportion of LTPA sites (prop.ltpa.sites)	0.005	0.05
Mean quality score of LTPA sites (mean.ql)	0	1

LTPA: leisure-time physical activity.
