# Supplementary material for: Exploring the emergence and evolution of population patterns of leisure-time physical activity through agent-based modelling
Source: Int J Behav Nutr Phys Act. 2018 Nov 19;15:112. doi: 10.1186/s12966-018-0750-9 (PMC6245872; doi:10.1186/s12966-018-0750-9)
Supplement: Supplementary file 3 — Summary of results of individual sensitivity analysis. (PDF 113 kb) [file 12966_2018_750_MOESM3_ESM.pdf]

Additional file 3 of "Exploring the emergence and evolution of population patterns of leisure-time physical activity through agent-based modelling", by Leandro M. T. Garcia, Ana V. Diez Roux, André C. R. Martins, Yong Yang, and Alex A. Florindo.

SUMMARY OF RESULTS OF INDIVIDUAL SENSITIVITY ANALYSIS

The table below summarizes the results expressed in 910 charts and 182 spreadsheets (65 charts and 13 spreadsheets for each of the 14 parameters) available at https://osf.io/j2kas/.

Table S3.1. Summary of results from the individual sensitivity analysis.

Parameter	% LTPA	% Low intention	% Intermediate intention	% High intention
Population				
Influence of the person's behavior in the previous week over his current intention (alpha.behavior)*	- Sensitivity: Low.<br>- A Test: Scores depart from 0.5 for parameter values lower than the reference (0.5014), more intensely for lower values and over time.<br>- Boxplots: In all years, similar proportions across all parameter values tested.	- Sensitivity: High.<br>- A Test: Until year 3, scores rapidly depart from 0.5 for parameter values diverging from the reference (0.5014), becoming stable by year 5, and decreasing from year 6 onwards, but still showing significant differences.<br>- Boxplots: Proportions increase as the parameter value increases.	- Sensitivity: High.<br>- A Test: Over time, scores rapidly depart from 0.5 as parameter values diverge from the reference (0.5014).<br>- Boxplots: Proportions and dispersions reduce as the parameter values increases, with larger reductions over time.	- Sensitivity: High.<br>- A Test: Until year 6, scores depart rapidly from 0.5 for parameters values diverging from the reference (0.5014), becoming stable by year 7, and decreasing by year 9, but still showing significant differences.<br>- Boxplots: Proportions increase as the parameter value increases.
Size of the person's perception radius (perception.radius)*	- Sensitivity: High.<br>- A Test: Scores depart from 0.5 for parameter values diverging from the reference (9), more intensely the further the value is.<br>- Boxplots: In all years, proportions increase rapidly for parameter values ranging from 5 to 11, stabilizing from then on.	- Sensitivity: High.<br>- A Test: Scores depart from 0.5 for parameter values diverging from the reference (9), more intensely the further the value is and over time.<br>- Boxplots: In all years, proportions decrease rapidly for parameter values ranging from 5 to 11, stabilizing from then on.	- Sensitivity: High.<br>- A Test: Scores depart from 0.5 for parameter values diverging from the reference (9), more intensely the further the value is, especially for lower values, and over time.<br>- Boxplots: Proportions increase rapidly for parameters values ranging from 5 to 11, and faster until year 4, stabilizing from then on.	- Sensitivity: High.<br>- A Test: Scores depart from 0.5 for parameter values diverging from the reference (9), more intensely the further the value is, especially for lower values, and over time.<br>- Boxplots: In all years, proportions increase rapidly for parameter values ranging from 5 to 11, stabilizing from then on.

continues

Table S3.1. Summary of results from the individual sensitivity analysis (*continuation*).

Parameter	% LTPA	% Low intention	% Intermediate intention	% High intention
Social environment				
Size of the proximal network (network.size)	Sensitivity: None or very low.	Sensitivity: None or very low.	- Sensitivity: Medium.<br>- A Test: Scores depart from 0.5 for parameter values lower than the reference (10), more intensely for values lower than 5 from year 4 onwards.<br>- Boxplots: Over time, proportions increase sharply for parameter values ranging from 2 to 10, stabilizing from then on.	Sensitivity: None or very low.
Proportion of people within the perception radius observed every week (observed.comm)	Sensitivity: None or very low.	Sensitivity: None or very low.	Sensitivity: None or very low.	Sensitivity: None or very low.
Influence of the proximal network's behavior over the person's intention (alpha.network)*	- Sensitivity: Low.<br>- A Test: Scores depart from 0.5 for parameter values lower than the reference (0.512), but not significantly.<br>- Boxplots: In all years, similar proportions across all parameter values tested.	- Sensitivity: Medium.<br>- A Test: Scores depart from 0.5 for parameter values lower than the reference (0.5012). From year 3 onwards, significant differences are found for values ≤ 0.5006.<br>- Boxplots: Over time, slight tendency of higher proportions as the parameter value increases.	- Sensitivity: Medium.<br>- A Test: Scores depart from 0.5 for parameter values diverging from the reference (0.512). From year 4 onwards, significant differences emerge, getting sharper until year 7, and stabilizing from year 8 onwards.<br>- Boxplots: Over time, slight tendency of lower proportions as the parameter value increases.	- Sensitivity: Low.<br>- A Test: Scores depart from 0.5 for parameter values lower than the reference (0.512), but not significantly.<br>- Boxplots: In all years, similar proportions across all tested values, but larger dispersions as the value increases.

continues

Table S3.1. Summary of results from the individual sensitivity analysis (*continuation*).

Parameter	% LTPA	% Low intention	% Intermediate intention	% High intention
Influence of the perceived community's behavior over the person's intention (alpha.comm)*	- Sensitivity: Low.<br>- A Test: Over time, scores depart from 0.5 for parameter values diverging from the reference (0.501), but not significantly.<br>- Boxplots: Over time, slight tendency of lower proportions as the parameter value increases.	- Sensitivity: Low.<br>- A Test: Over time, scores depart from 0.5 for parameter values diverging from the reference (0.501), but not significantly.<br>- Boxplots: Over time, slight tendency of higher proportions and dispersions as the parameter value increases.	- Sensitivity: Low.<br>- A Test: Over time, scores depart from 0.5 for parameter values diverging from the reference (0.501), but not significantly.<br>- Boxplots: Over time, slight tendency of lower proportions as the parameter value increases.	- Sensitivity: Low.<br>- A Test: Over time, scores depart from 0.5 for parameter values diverging from the reference (0.501), but not significantly.<br>- Boxplots: Over time, slight tendency of lower proportions and higher dispersions as the parameter value increases.
Built environment				
Proportion of LTPA sites (prop.ltpa.sites)*	- Sensitivity: High.<br>- A Test: Scores depart from 0.5 for parameter values diverging from the reference (1.5).<br>- Boxplots: In all years, proportions increase rapidly for parameter values ranging from 0.5 to 2.5, stabilizing from then on.	- Sensitivity: High.<br>- A Test: Until year 3, scores depart from 0.5 for parameter values diverging from the reference (1.5), stabilizing from then on.<br>- Boxplots: In all years, proportions decrease rapidly for parameter values ranging from 0.5 to 2.5, stabilizing from then on.	- Sensitivity: High.<br>- A Test: Until year 5, scores depart from 0.5 for parameter values diverging from the reference (1.5), especially for lower values, stabilizing from year 6 onwards.<br>- Boxplots: In all years, proportions increase rapidly for parameter values ranging from 0.5 to 2.5, stabilizing from then on.	- Sensitivity: High.<br>- A Test: Until year 5, scores depart from 0.5 for parameter values diverging from the reference (1.5), stabilizing from year 6 onwards.<br>- Boxplots: In all years, proportions increase rapidly for parameter values ranging from 0.5 to 2.5, stabilizing from then on in year 5.
Minimum number of activities available in LTPA sites (min.activities)	Sensitivity: None or very low.	Sensitivity: None or very low.	Sensitivity: None or very low.	Sensitivity: None or very low.
Maximum number of activities available in LTPA sites (max.activities)	Sensitivity: None or very low.	Sensitivity: None or very low.	Sensitivity: None or very low.	Sensitivity: None or very low.

continues

Table S3.1. Summary of results from the individual sensitivity analysis (*continuation*).

Parameter	% LTPA	% Low intention	% Intermediate intention	% High intention
Mean quality score of LTPA sites (mean.ql)*	- Sensitivity: Low.<br>- A Test: Scores depart from 0.5 for parameter values lower than the reference (0.5), but not significantly.<br>- Boxplots: In all years, similar proportions among all parameter values tested.	- Sensitivity: Low.<br>- A Test: Scores depart from 0.5 for parameter values lower than the reference (0.5), but not significantly.<br>- Boxplots: Over time, slight tendency of higher proportions for parameter values lower than 0.5.	- Sensitivity: Low.<br>- A Test: Scores depart from 0.5 for parameter values lower than the reference (0.5), but not significantly.<br>- Boxplots: In all years, similar proportions among all parameter values tested.	- Sensitivity: Low.<br>- A Test: Scores depart from 0.5 for parameter values lower than the reference (0.5), but not significantly.<br>- Boxplots: Over time, slight tendency of higher proportions as the parameter value increases.
Standard deviation of quality score of LTPA sites (sd.ql)	Sensitivity: None or very low.	Sensitivity: None or very low.	Sensitivity: None or very low.	Sensitivity: None or very low.
Mean of scaling factor representing the persons' subjective assessment of LTPA sites (mean.perception)	Sensitivity: None or very low.	Sensitivity: None or very low.	Sensitivity: None or very low.	Sensitivity: None or very low.
Standard deviation of scaling factor representing the persons' subjective assessment of LTPA sites (sd.perception)	Sensitivity: None or very low.	Sensitivity: None or very low.	Sensitivity: None or very low.	Sensitivity: None or very low.
Scaling factor of the built environment's influence (r)	Sensitivity: None or very low.	Sensitivity: None or very low.	Sensitivity: None or very low.	Sensitivity: None or very low.

%: proportion of [*output*]. LTPA: leisure-time physical activity. *: parameters selected for the global sensitivity analysis.
