# Supplementary material for: Exploring the emergence and evolution of population patterns of leisure-time physical activity through agent-based modelling
Source: Int J Behav Nutr Phys Act. 2018 Nov 19;15:112. doi: 10.1186/s12966-018-0750-9 (PMC6245872; doi:10.1186/s12966-018-0750-9)
Supplement: Supplementary file 4 — Results of global sensitivity analysis (year 10). (PDF 712 kb) [file 12966_2018_750_MOESM4_ESM.pdf]

Additional file 4 of "Exploring the emergence and evolution of population patterns of leisure-time physical activity through agent-based modelling", by Leandro M. T. Garcia, Ana V. Diez Roux, André C. R. Martins, Yong Yang, and Alex A. Florindo.

RESULTS OF GLOBAL SENSITIVITY ANALYSIS (YEAR 10)

The graphs below summarize the correlation between selected model parameters and outputs using data obtained at the 520th iteration (equivalent to end of year 10). Results for every year are expressed in 325 charts and 26 spreadsheets available at https://osf.io/j2kas/.

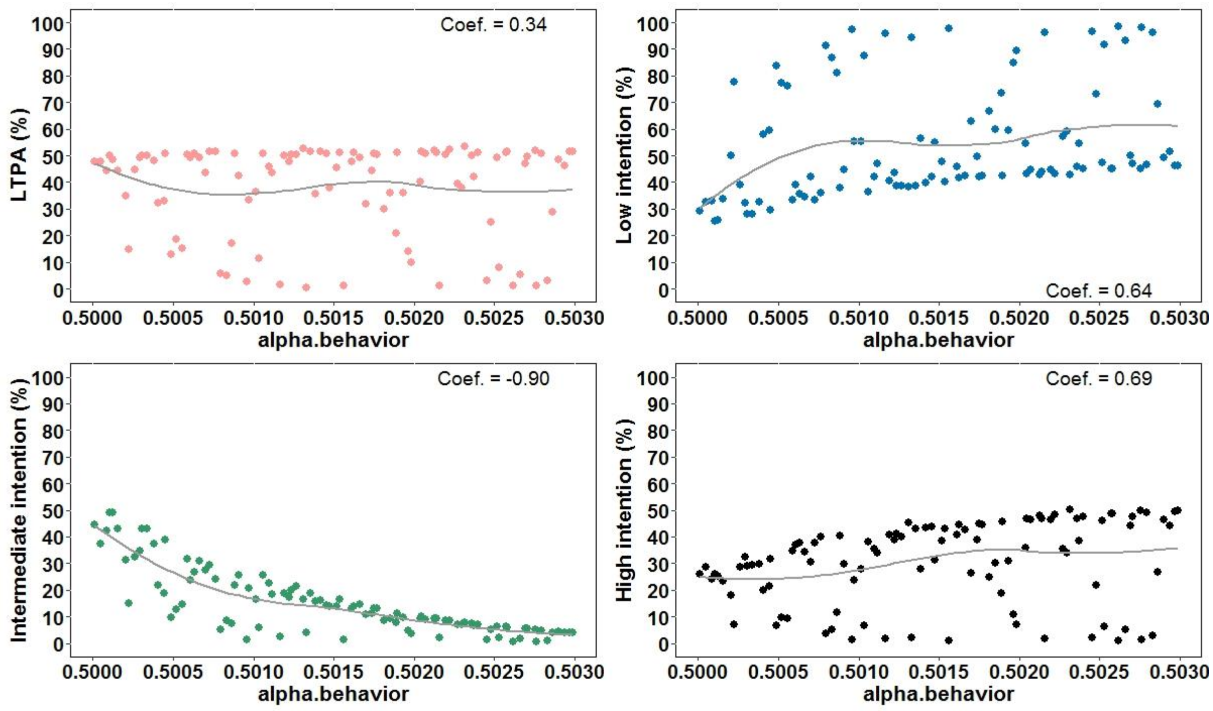

Figure S4.1. Correlation between the influence of the person's behavior in the previous week on his current intention (alpha.behavior) and the model's outputs. Obtained at the 520th iteration (equivalent to end of year 10). Outputs: red = proportion of people practicing leisure-time physical activity (LTPA); blue = proportion of people with low intention (0.03 ≤ intention < 0.25); green = proportion of people with intermediate intention (0.25 ≤ intention ≤ 0.75); black = proportion of people with high intention (0.75 < intention ≤ 0.97).

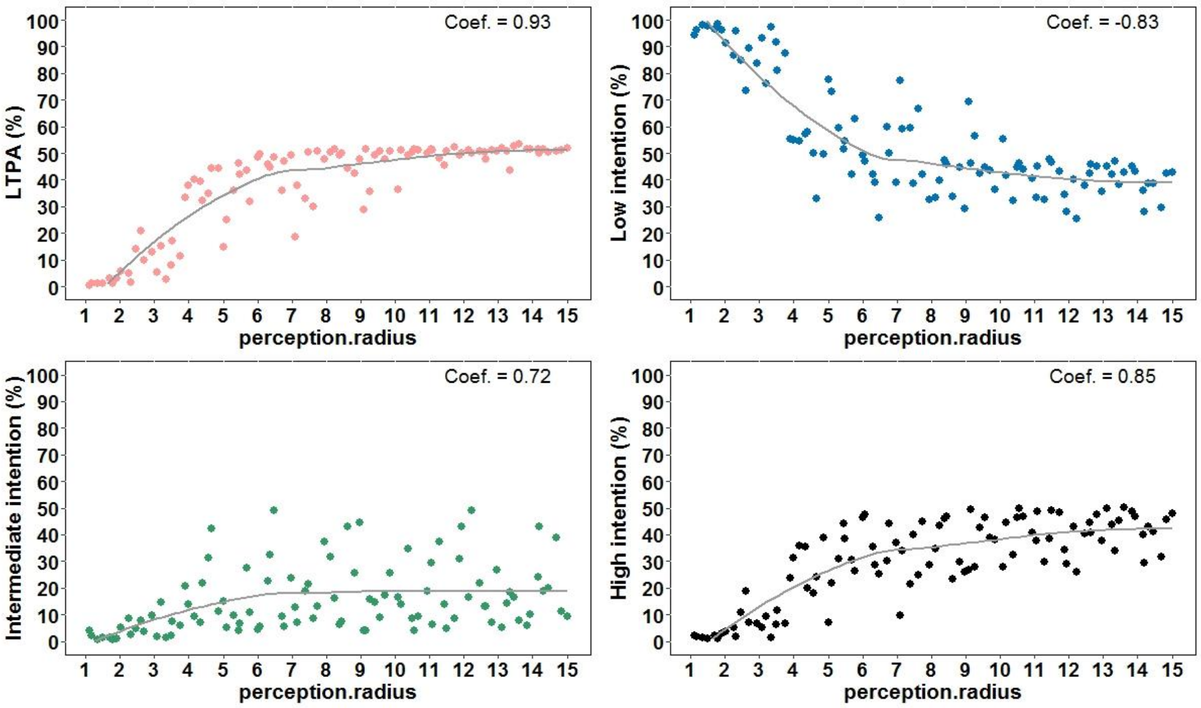

Figure S4.2. Correlation between the size of the persons' perception radius (perception.radius) and the model's outputs. Obtained at the 520th iteration (equivalent to end of year 10). Outputs: red = proportion of people practicing leisure-time physical activity (LTPA); blue = proportion of people with low intention (0.03 ≤ intention < 0.25); green = proportion of people with intermediate intention (0.25 ≤ intention ≤ 0.75); black = proportion of people with high intention (0.75 < intention ≤ 0.97).

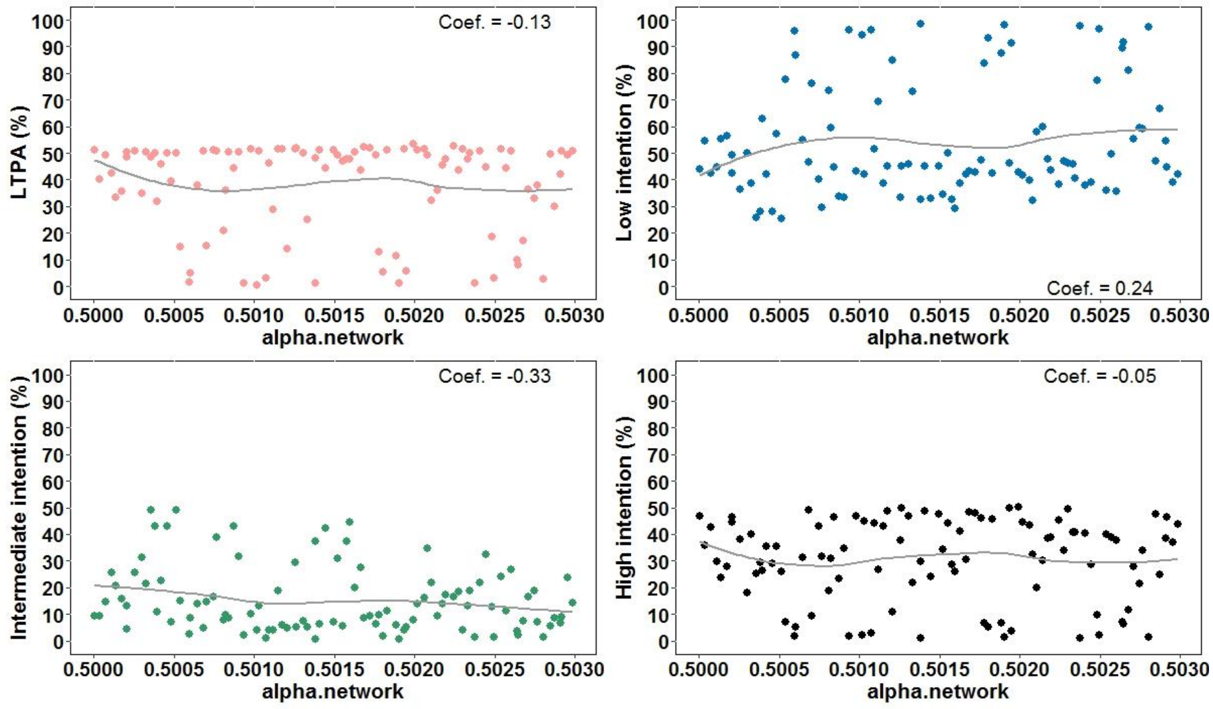

Figure S4.3. Correlation between the influence of the proximal network's behavior on person's intention (alpha.network) and the model's outputs. Obtained at the 520th iteration (equivalent to end of year 10). Outputs: red = proportion of people practicing leisure-time physical activity (LTPA); blue = proportion of people with low intention ($0.03 \leq$ intention < 0.25); green = proportion of people with intermediate intention ($0.25 \leq$ intention ≤ 0.75); black = proportion of people with high intention ($0.75 <$ intention ≤ 0.97).

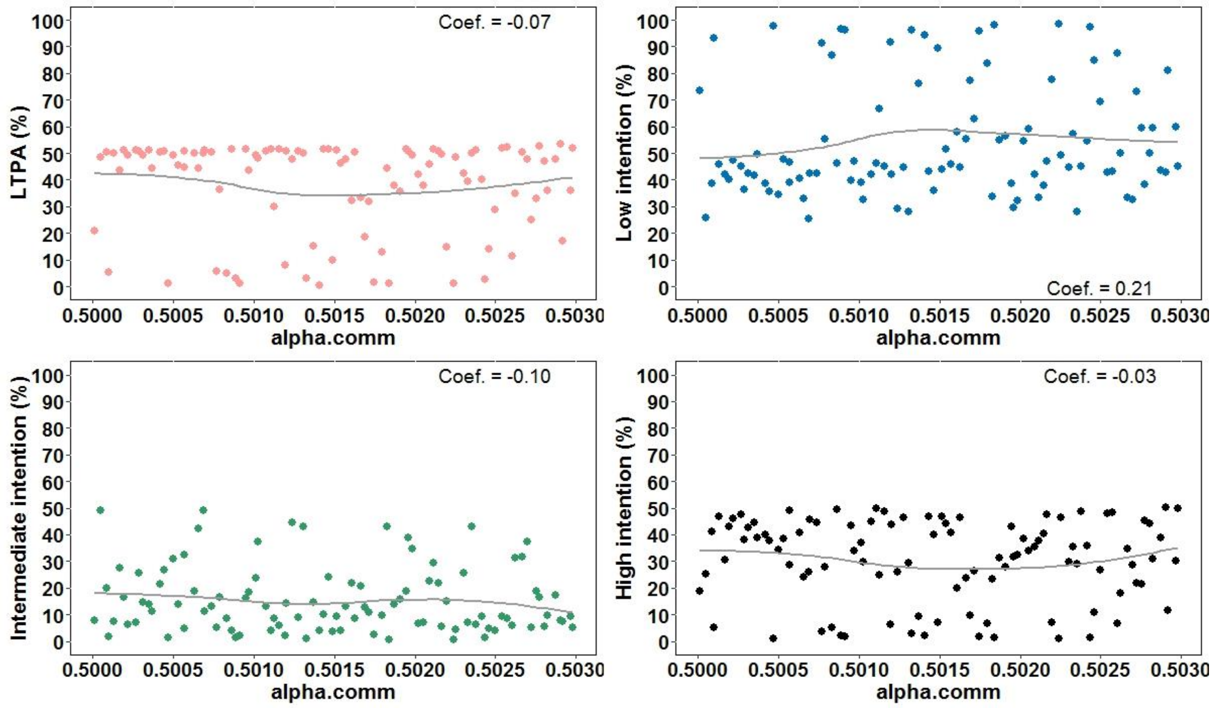

Figure S4.4. Correlation between the influence of the perceived community's behavior on person's intention (alpha.comm) and model's outputs. Obtained at the 520th iteration (equivalent to end of year 10). Outputs: red = proportion of people practicing leisure-time physical activity (LTPA); blue = proportion of people with low intention (0.03 ≤ intention < 0.25); green = proportion of people with intermediate intention (0.25 ≤ intention ≤ 0.75); black = proportion of people with high intention (0.75 < intention ≤ 0.97).

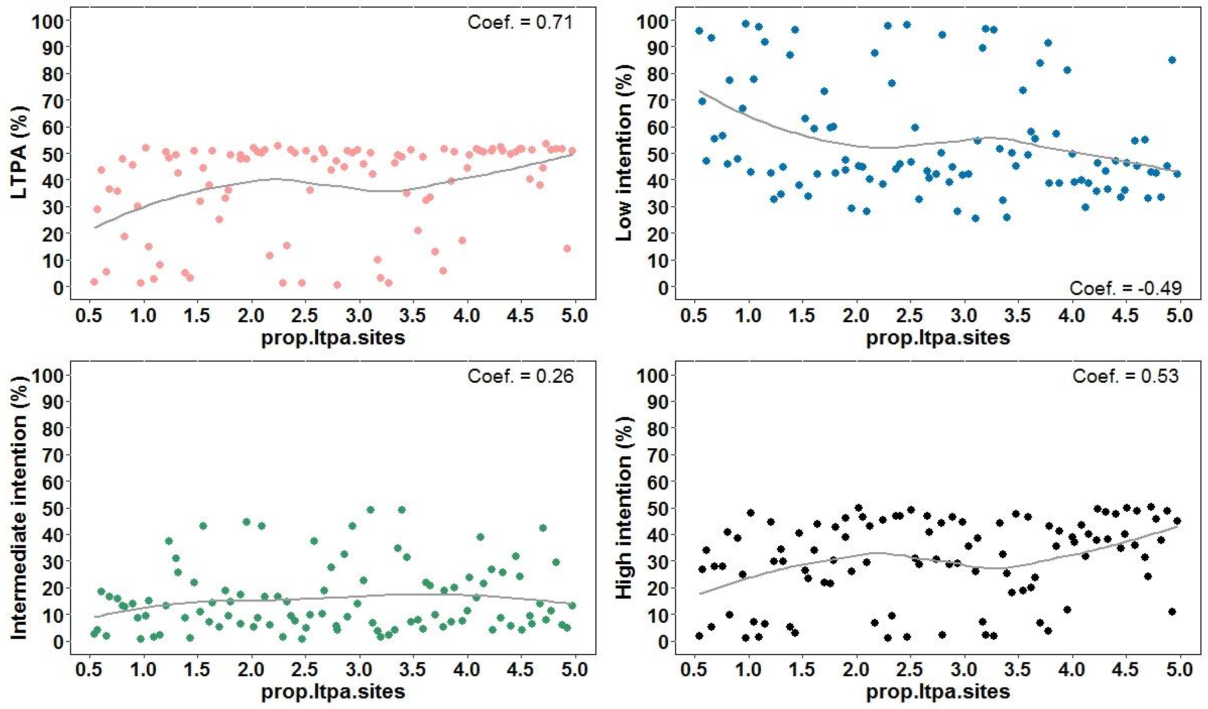

Figure S4.5. Correlation between the proportion of leisure-time physical activity (LTPA) sites (prop.ltpa.sites) and model's outputs. Obtained at the 520th iteration (equivalent to end of year 10). Outputs: red = proportion of people practicing LTPA; blue = proportion of people with low intention (0.03 ≤ intention < 0.25); green = proportion of people with intermediate intention (0.25 ≤ intention ≤ 0.75); black = proportion of people with high intention (0.75 < intention ≤ 0.97).

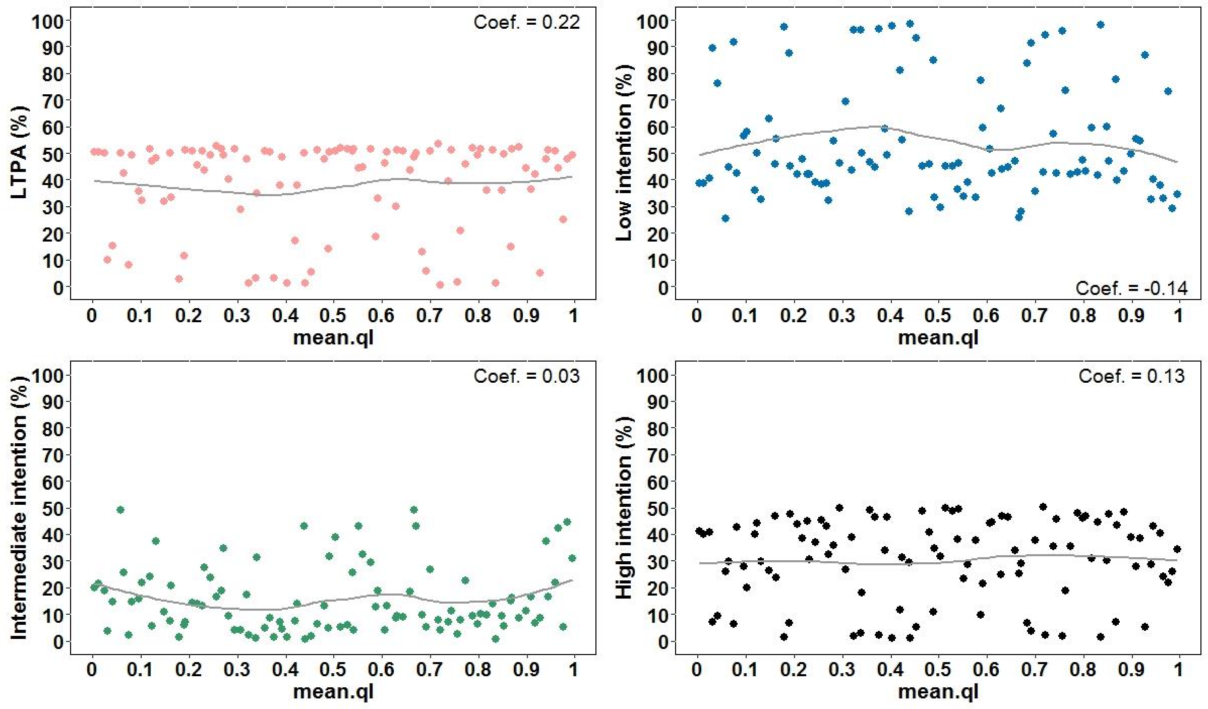

Figure S4.6. Correlation between the mean quality score of leisure-time physical activity (LTPA) sites (mean.ql) and model's outputs. Obtained at the 520th iteration (equivalent to end of year 10). Outputs: red = proportion of people practicing LTPA; blue = proportion of people with low intention ($0.03 \leq$ intention < 0.25); green = proportion of people with intermediate intention ($0.25 \leq$ intention ≤ 0.75); black = proportion of people with high intention ($0.75 <$ intention ≤ 0.97).
